# Performance of Graphene–CdS Hybrid Nanocomposite Thin Film for Applications in Cu(In,Ga)Se_2_ Solar Cell and H_2_ Production

**DOI:** 10.3390/nano10020245

**Published:** 2020-01-30

**Authors:** Salh Alhammadi, Vasudeva Reddy Minnam Reddy, Sreedevi Gedi, Hyeonwook Park, Mostafa Saad Sayed, Jae-Jin Shim, Woo Kyoung Kim

**Affiliations:** School of Chemical Engineering, Yeungnam University, Gyeongsan, Gyeongbuk 38541, Korea; salehalhammadi.1987@gmail.com (S.A.); drmvasudr9@gmail.com (V.R.M.R.); drsrvi9@gmail.com (S.G.); greatekal@naver.com (H.P.); mostafassayed@gmail.com (M.S.S.); jjshim@yu.ac.kr (J.-J.S.)

**Keywords:** Gr–CdS, cadmium sulfide, Cu(In, Ga)Se_2_, buffer layer, water splitting

## Abstract

A graphene–cadmium sulfide (Gr–CdS) nanocomposite was prepared by a chemical solution method, and its material properties were characterized by several analysis techniques. The synthesized pure CdS nanoparticles (NPs) and Gr–CdS nanocomposites were confirmed to have a stoichiometric atomic ratio (Cd/S = 1:1). The Cd 3d and S 2p peaks of the Gr–CdS nanocomposite appeared at lower binding energies compared to those of the pure CdS NPs according to X-ray photoelectron spectroscopy analyses. The formation of the Gr–CdS nanocomposite was also evidenced by the structural analysis using Raman spectroscopy and X-ray diffraction. Transmission electron microscopy confirmed that CdS NPs were uniformly distributed on the graphene sheets. The absorption spectra of both the Gr–CdS nanocomposite and pure CdS NPs thin films showed an absorption edge at 550 nm related to the energy band gap of CdS (~2.42 eV). The Cu(In,Ga)Se_2_ thin film photovoltaic device with Gr–CdS nanocomposite buffer layer showed a higher electrical conversion efficiency than that with pure CdS NPs thin film buffer layer. In addition, the water splitting efficiency of the Gr–CdS nanocomposite was almost three times higher than that of pure CdS NPs.

## 1. Introduction

In recent years, cadmium sulfide (CdS) semiconductor has attracted attention for various applications because of its wide energy band gap (~2.42 eV), high transparency, high stability, n-type conductivity, emission tunability, high extinction coefficient, large dipole moment, and potential sensitization [1,2,3,4]. CdS thin films have been widely used as n-type buffer layers in high-efficiency Cu(In, Ga)Se_2_ (CIGS) thin film solar cells with the structure of glass/Mo/CIGS/CdS/i-ZnO/ZnO:Al(AZO) [5,6,7], where the p-n junction is formed at the interface of CIGS/CdS. In addition, CdS has attracted great attention as a photoanode for photocatalytic water splitting application [8,9], where it can absorb visible light and promote the separation of photogenerated carriers [8].

Conventionally, the optoelectronic properties of CdS can be tailored by two approaches, i.e., doping and compositing. In the doping process, the various dopants such as aluminum [10], copper [11], nickel [12], sodium [13], and gallium [14] have been employed, where the lattice of the host (CdS) can be changed by the dopant atoms [15]. In compositing, CdS is combined with one or more materials to obtain unique and improved material properties. For example, the intrinsic properties of graphene (Gr) and CdS nanoparticles (NPs) have been combined in nanocomposites to produce a photocurrent from the visible light. Furthermore, because graphene involves π conjugation, it exhibits unique emergent electrical, mechanical, and thermal properties. It possesses high mobility, flexibility, and stability. The electrons in graphene behave as massless fermions due to the linear relationship between the energy and the momentum, where the electronic properties are determined by the Dirac equation; consequently, it has potential for use in optoelectronic applications [16,17,18].

Recently, graphene and its derivatives have been applied to a variety of solar cells as a front/back electrode and other functional layer, as summarized in Table 1.

In addition, these unique properties of graphene make it compatible with host CdS NPs, which decorate the surface of graphene in graphene-cadmium sulfide (Gr–CdS) nanocomposites. Compared to the pure CdS NPs, the electrical properties of Gr–CdS were reported to be greatly enhanced [35,36,37]. There were many reports on the photoelectrochemical and photocatalytic applications of Gr-CdS [35,38]. However, Gr–CdS composites have not yet been employed as a buffer layer in CIGS photovoltaic devices.

Therefore, in this work, the Gr–CdS composite and CdS NPs were fabricated by chemical solution method, and their properties were investigated for CIGS photovoltaic device as well as photocatalytic application. The performance of CIGS photovoltaic devices with the Gr–CdS composite as a buffer layer was compared with that of CIGS photovoltaic devices with a conventional CdS buffer layer. Furthermore, the photoelectrocatalytic (PEC) water splitting efficiency of the Gr-CdS composite was compared to that of pure CdS NPs.

## 2. Materials and Methods 

### 2.1. Synthesis of CdS NPs and Gr–CdS Nanocomposite

To prepare the CdS NPs and Gr–CdS nanocomposite using a cost-effective chemical precipitation method, 0.1 M cadmium sulfate (CdSO_4_), 0.02 M thiourea [SC(NH_2_)_2_], and 100 mL of a graphene aqueous solution were used. Graphene aqueous solution was prepared by dispersing 0.2 g of graphene sheets in 100 mL of deionized (DI) water and subjecting this to ultrasonication. The 7~8 layer graphene sheets (ILJIN Nano Tech, Seoul, South Korea) with a mean length of 500 nm were used. First, CdS NPs were synthesized by mixing 0.1 M cadmium sulfate and 0.02 M thiourea at a constant pH of 10 (which was maintained using NH_4_OH). Next, the Gr–CdS nanocomposite was synthesized by adding 0.1 M cadmium sulfate and 0.02 M thiourea to 100 mL of the graphene aqueous solution at a constant pH of 10 (which was maintained as described above). Both the CdS NPs and Gr–CdS nanocomposites were synthesized at room temperature for a reaction time of 2 h. Furthermore, the obtained CdS NPs and Gr–CdS nanocomposites were centrifuged and rinsed several times using DI water and ethanol. Finally, the CdS NPs and Gr–CdS nanocomposites were dried in a convection oven at 60 °C for 24 h.

### 2.2. Film Preparation Using CdS NPs and Gr–CdS Nanocomposite

**Figure 1:**
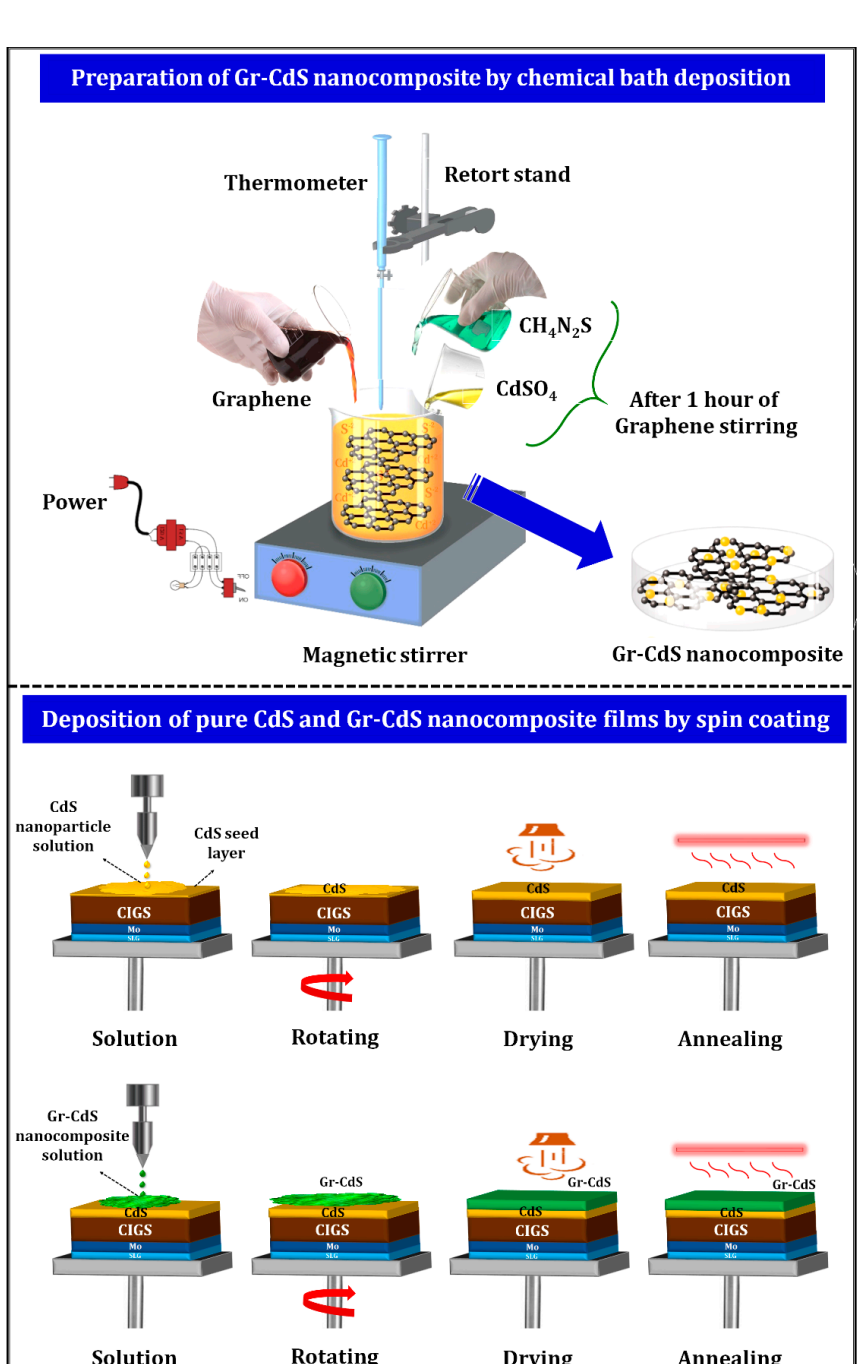
Schematic of the deposition of CdS nanoparticles (NPs) and Gr–CdS nanocomposite films.

Two substrates, bare soda-lime glass (SLG) and molybdenum sandwiched between SLG and CIGS (which is referred to as a CIGS-coated substrate) were used to prepare the CdS NPs and Gr–CdS nanocomposite films. The SLG substrates were used to study the optical properties, structural properties, and chemical state of the NPs, and the CIGS-coated substrates were used to prepare the photovoltaic device. Prior to the deposition of CdS NPs and Gr–CdS nanocomposite films, a seed layer consisting of a CdS thin film was coated by chemical bath deposition (CBD) to enhance the deposition of the CdS NPs and Gr–CdS nanocomposite films by spin-coating. Details on the deposition of the CdS films by CBD are given in our previous report [14]. To spin-coat the pure CdS NPs and Gr–CdS nanocomposite films, inks consisting of pure CdS NPs and the Gr–CdS nanocomposite were prepared by dissolving 0.05 g of pure CdS NPs or the Gr–CdS nanocomposite in 10 mL of absolute ethanol and sonicating the solution for 15 min to obtain homogenous deposition. The prepared ink was spin-coated on the substrates at 3000 rpm. The entire experimental procedure is schematically illustrated in Figure 1.

### 2.3. CIGS Device Fabrication 

Photovoltaic devices with a structure of glass/Mo/CIGS/CdS NPs (or Gr–CdS)/i-ZnO/AZO/Ni:Al were used, where the i-ZnO/AZO transparent conducting oxide layers were deposited by sputtering method and the Ni/Ag front grids were added by electron-beam (E-beam) evaporation.

### 2.4. Characterization 

#### 2.4.1. Material Characterization

The crystal structures and phases were analyzed using X-ray diffraction (XRD) (X’Pert PRO-Multipurpose Diffractor, PANalytical, The Netherlands) using Cu Kα radiation (λ = 0.15406 nm). The microstructure was observed by transmission electron microscopy (TEM) (Tecnai G2 F20 S-TWIN, Thermo Fisher Scientific, The Netherlands) at an accelerating voltage of 200 kV. Selected-area electron diffraction (SAED) analysis was also conducted using TEM to determine the interplanar distance and crystalline phase of the Gr–CdS nanocomposite. Raman spectroscopy (XploRA Plus, Horiba, Japan) with 532nm laser source was performed to further confirm the phase and structure. The elemental composition was obtained by energy-dispersive spectrometry (EDS) (S-4200, HITACHI, Japan). X-ray photoelectron spectroscopy (XPS) (K-Alpha, Thermo Fisher Scientific, UK) was employed to investigate the chemical state of the CdS NPs and Gr–CdS nanocomposite. The optical properties were examined by UV–vis–NIR spectroscopy (Cary 5000, Agilent, USA).

#### 2.4.2. Solar Cell Characterization

To evaluate the photovoltaic performance of the fabricated CIGS solar cells, the illuminated current density–voltage (J–V) characteristics under AM 1.5 one-sun conditions were measured using an AAA-class solar simulator (K201, LAB 55, McScience, South Korea).

#### 2.4.3. PEC Measurement

To investigate the photoelectrochemical properties of the prepared materials, a three-electrode PEC cell with a quartz window was used. In the PEC cell, 0.5 M of Na_2_SO_4_ purged with nitrogen was employed as electrolyte. Aqueous slurries of pure CdS NPs or Gr–CdS (50 μL) were spread over a cleaned fluorine-doped tin oxide glass substrate to prepare the photoanodes. The prepared photoanodes, Pt flag and Ag/AgCl were used as working, counter, and reference electrodes, respectively. The suspension was prepared by dispersing pure CdS NPs or Gr–CdS (20 mg) with a Nafion solution in ethanol (1 wt%, 2 mL). In order to illuminate the working electrodes with visible light, a solar simulator light (Abet-10500, 150 W) with an intensity of 100 mW/cm^2^ was used. All the PEC measurements as linear sweep voltammetry (LSV), chronoamperometry, and electrochemical impedance spectroscopy (EIS) were performed using an electrochemical potentiostat (PGSTAT 302N, Autolab, The Netherlands) connected to the PEC cell. The measured potential vs. the Ag/AgCl reference electrode was converted to the reversible hydrogen electrode (RHE) by the Nernst equation

ERHE = EAg/AgCl + 0.0591 × pH + 0.1976(1)

## 3. Results

### 3.1. Properties of CdS NPs and Gr–CdS Nanocomposite

#### 3.1.1. EDS Analysis

Table 2 shows the elemental composition determined by EDS for the pure CdS NPs and Gr–CdS nanocomposite. The composition ratio of Cd/S in the pure CdS NPs and Gr–CdS is 0.91–0.93. However, in the Gr–CdS nanocomposite, 77.8 at% of carbon was detected due to the presence of graphene.

#### 3.1.2. XPS Analysis

**Figure 2:**
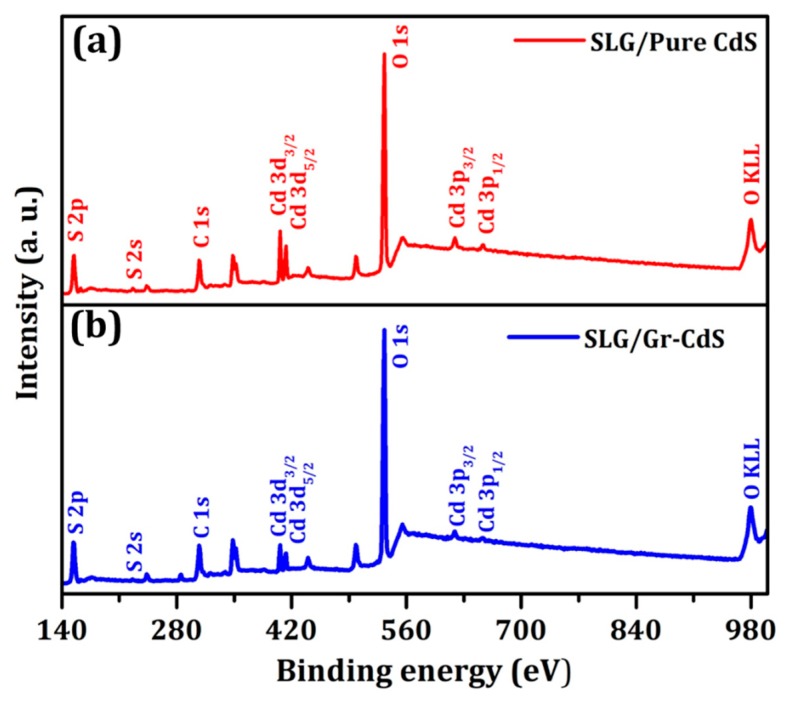
XPS survey spectra of (**a**) pure CdS NPs and (**b**) Gr–CdS nanocomposite.

**Figure 3:**
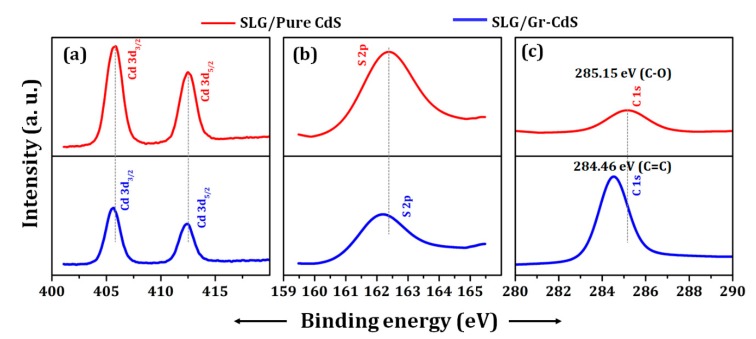
High-resolution XPS spectra of pure CdS NPs and Gr–CdS nanocomposite: (**a**) Cd 3d, (**b**) S 2p, and (**c**) C 1s.

To confirm the formation of Gr–CdS nanocomposite, XPS analysis was adopted. Figure 2 and Figure 3 show the XPS of the pure CdS NPs and Gr–CdS nanocomposite. As seen from the survey spectra of Figure 2, the characteristic Cd 3d, S 2p, C 1s, and O 1s peaks are confirmed. The high-resolution spectra in Figure 3a show two peaks at 405 and 412 eV which are assigned to the 3d_5/2_ and 3d_3/2_ of Cd^2+^ in CdS [14]. The binding energy difference between the Cd 3d_5/2_ and 3d_3/2_ peaks is approximately 7 eV for the pure CdS and Gr–CdS nanocomposite, which further confirms that Cd has the +2 oxidation state [39,40]. Figure 3b shows peaks at 162.22 and 161.98 eV corresponding to the S^2-^ peak of CdS [39,40]. As shown in Figure 2a,b, the Cd 3d and S 2p peak intensities of the Gr–CdS nanocomposite are lower than those of the pure CdS NPs; this result indicates a decrease in the atomic composition of Cd and S after the formation of Gr–CdS nanocomposite, which is consistent with the results of EDS analysis in Table 2. As shown in Figure 3c, the Gr–CdS nanocomposite exhibited a strong C 1s peak at a binding energy of 284.46 eV, which corresponds to sp^2^ hybridized carbon in graphene, whereas the CdS NPs exhibited a relatively weak C 1s peak at 285.15 eV, which corresponds to C–O [41,42].

#### 3.1.3. XRD Analysis

**Figure 4:**
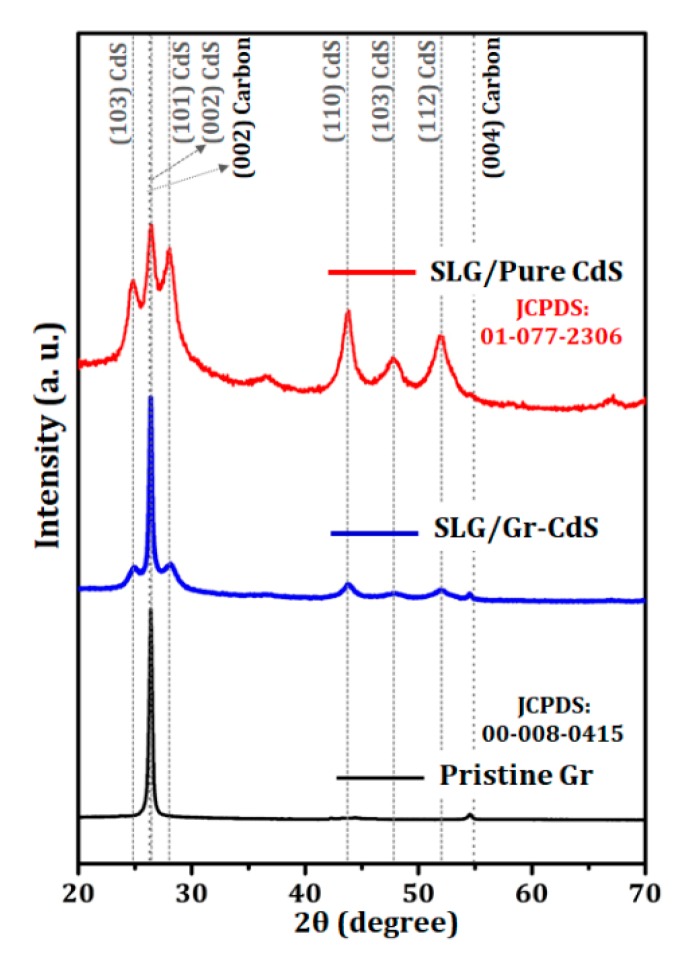
XRD results of pristine graphene, Gr–CdS nanocomposite, and pure CdS NPs.

Figure 4 shows the XRD patterns of the pristine graphene, pure CdS NPs, and Gr–CdS nanocomposite. Pristine graphene powder was used as a reference for the XRD analysis of the Gr–CdS nanocomposite. The diffraction peaks of the pristine graphene at 2θ ~ 26° and 54° matched well those of graphene with hexagonal crystal structure (JCPDS No. 00-008-0415) and were indexed as the (002) and (004) facets, respectively [39,43]. In addition, all the XRD peaks of the pure CdS NPs matched well those of CdS with hexagonal crystal structure (JCPDS No. 00-001-0780) and were identified as the (100), (002), (101), (102), (110), (102), and (112) planes of the hexagonal structure [44]. Interestingly, the XRD pattern of the Gr–CdS nanocomposite appears to be a simple combination of the reflection patterns of the pristine graphene and pure CdS, where no significant changes appear in the relative intensity of each peak. It is believed that the most intense peak, at 2θ ~ 26°, corresponds to both carbon (002) and CdS (002), which appear at nearly identical positions. Overall, the XRD results suggest that the Gr–CdS nanocomposite is formed by physical mixing rather than by chemical bonding between the graphene and CdS NPs.

#### 3.1.4. Raman Analysis

**Figure 5:**
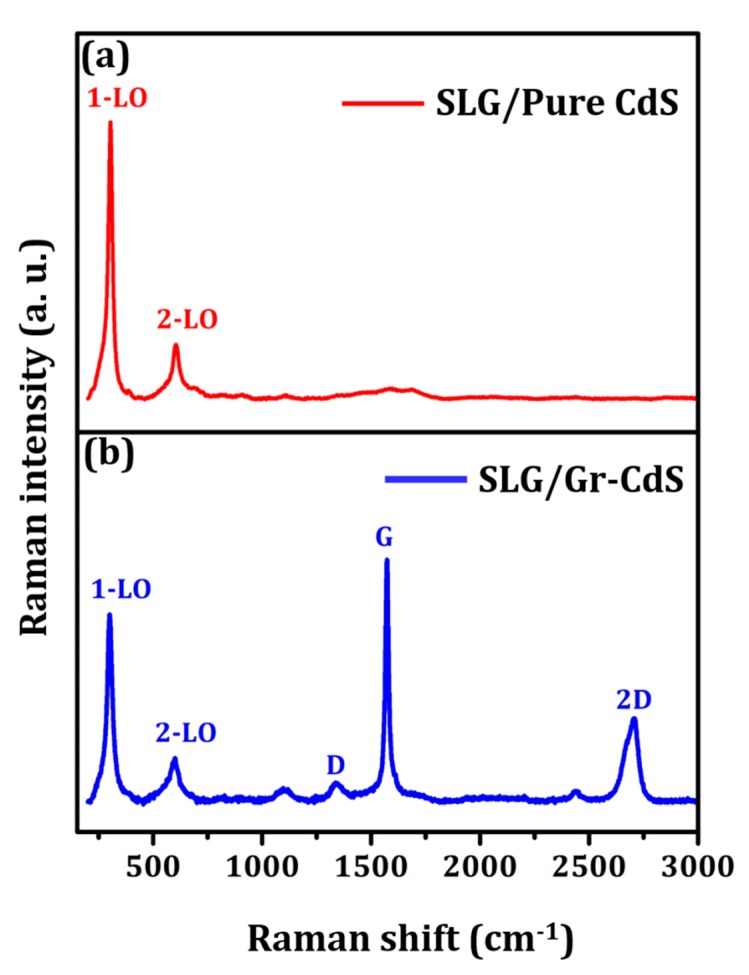
Raman spectra of (**a**) pure CdS NPs and (**b**) Gr–CdS nanocomposite.

Raman spectra for both the pure CdS NPs and Gr–CdS nanocomposite are shown in Figure 5. For the pure CdS NPs, the two peaks at 300 and 600 cm^−1^ were related to the longitudinal optical (LO) phonon modes of CdS. The vibrational mode at 300 cm^−1^ is the fundamental band (1LO), and that at 600 cm^−1^ is the overtone (2LO) [45]. For the Gr–CdS nanocomposite, three additional characteristic bands located at 1573, 1345, and 2711 cm^−1^ were observed, which correspond to the conventional G, D, and 2D peaks of graphene, respectively. The presence of the 2D band at approximately 2711 cm^−1^ indicates the multilayer nature of graphene, which was observed by other groups [46,47]. Furthermore, the very low intensity of the D peak indicates low disorder in the graphene structure [48,49]. However, the LO and 1LO peaks of the Gr–CdS nanocomposite were lower because of the lower CdS concentration compared to the pure CdS NPs. The above results confirmed the formation of the Gr–CdS nanocomposite.

#### 3.1.5. TEM Analysis

**Figure 6:**
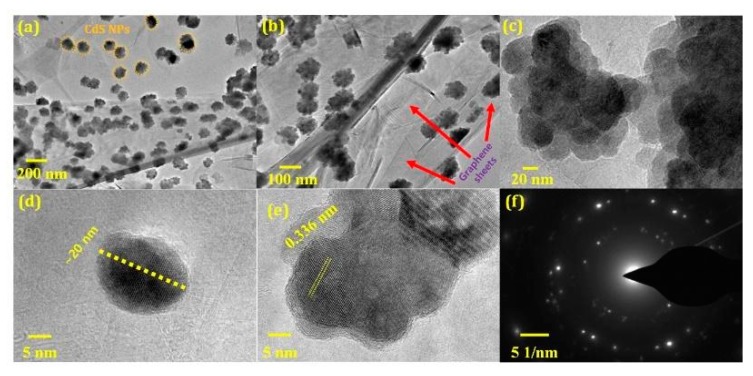
TEM images of (**a**,**b**) graphene sheets decorated with CdS NPs in Gr–CdS nanocomposite, (**c**) CdS NPs on the sheet, (**d**) crystal size, (**e**) lattice fringes, and (**f**) SAED pattern.

The morphological features and lattice parameters of the samples were determined by TEM, high-resolution TEM (HR-TEM), and SAED. Figure 6a,b clearly show that the graphene sheets are decorated by scattered CdS NPs. The corrugations in the Gr–CdS composite are a characteristic feature of graphene under the positional stress of the NPs [50,51]. In addition, aggregation of CdS NPs on the graphene sheet was also identified in Figure 6c. Furthermore, the average size of the CdS crystals was measured to be approximately 20–30 nm, as representatively shown in Figure 6d, whereas Figure 6e shows clear lattice fringes of CdS with a spacing of approximately 0.336 nm related to (002) plane of the hexagonal CdS, in agreement with the XRD analysis. In addition, a set of rings appears in Figure 6f, indicating that the Gr–CdS composite is polycrystalline.

#### 3.1.6. Optical Analysis 

**Figure 7:**
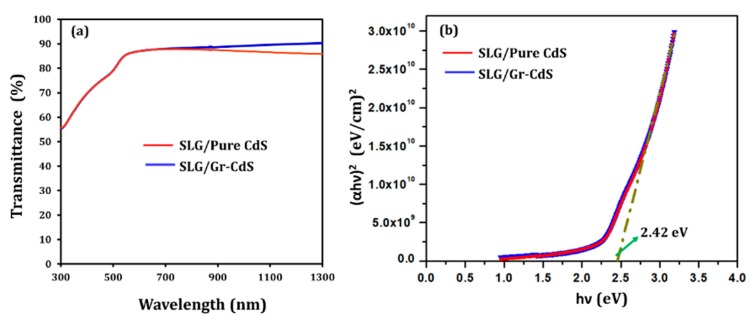
(**a**) Transmittance spectra and (**b**) (αhν)^2^ vs. hν plot of SLG/CdS and SLG/Gr–CdS thin films.

Figure 7 shows the optical transmittance and corresponding (αhν)^2^ vs. hν plots of the SLG/CdS and SLG/Gr–CdS films measured by UV–vis–NIR spectrometry. The Gr–CdS film exhibited a slight improvement (~5%) in transmittance and a decrease in absorbance, particularly at long wavelengths (greater than ~800 nm) owing to the presence of graphene. It is well known that graphene is highly transparent; thus, it was proposed as a front electrode in photovoltaic applications [22,30]. Similar optical behavior was reported for Pt NP/Gr thin films [52]. However, in the visible region, the transmittance of the Gr–CdS film was almost the same as that of the pure CdS thin film, which can probably be attributed to absorption of light by CdS NPs dispersed on the surface of the graphene sheets. Both films had an absorption edge near 550 nm, which agrees with the band gap of CdS (~2.42 eV) [14].

### 3.2. Applications of Pure CdS and SLG/Gr–CdS 

#### 3.2.1. CIGS Photovoltaic Devices

**Figure 8:**
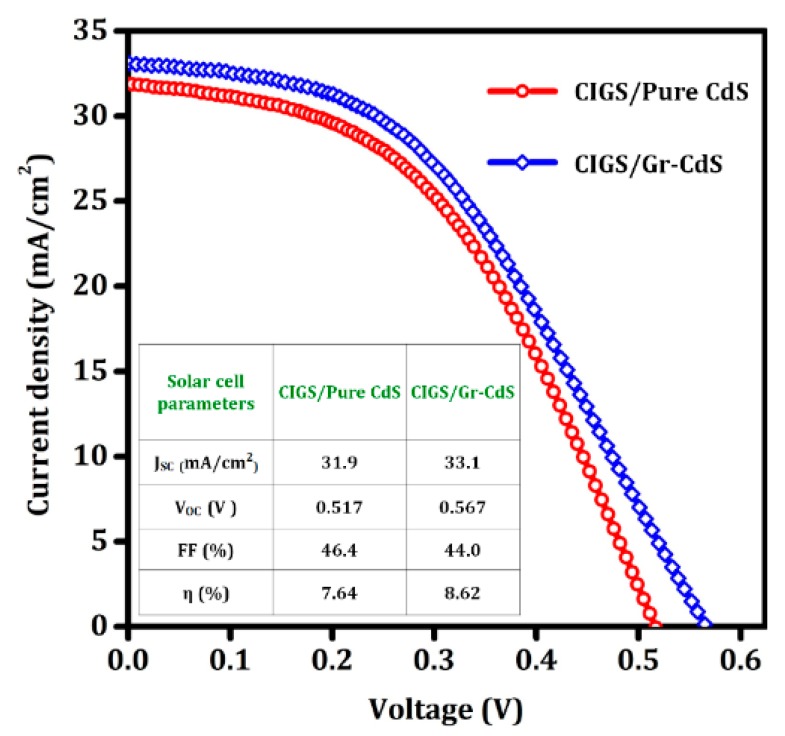
Current density–voltage characteristics of the CIGS/CdS and CIGS/Gr–CdS solar cells.

Using the pure CdS NPs and Gr–CdS nanocomposite as the buffer layer, CIGS photovoltaic devices were fabricated. The J–V characteristics of corresponding devices are compared in Figure 8. The CIGS device prepared with the Gr–CdS nanocomposite buffer layer showed better efficiency (with higher short-circuit current (J_SC_), and higher open-circuit voltage (V_OC_)) than that with the pure CdS NPs buffer layer, which may be partly due to the high electron mobility of the Gr–CdS nanocomposite. However, the fill factor (FF) of the CIGS photovoltaic device fabricated with the Gr–CdS buffer layer was slightly lower, presumably because of pinholes or surface inhomogeneities in the Gr–CdS nanocomposite films [53].

The enhanced device performance of the Gr–CdS-nanocomposite-based CIGS cell was driven by the high electron mobility of graphene and high photocurrent generation in the Gr–CdS nanocomposite thin film. Owing to the high electron mobility, the electrons generated in the CIGS absorber can be rapidly transported to the CIGS/Gr–CdS junction and then to the front contact without recombination, resulting in high current collection. It has been widely reported that the performance of semiconductor devices was enhanced significantly when graphene was combined with a semiconductor material. For example, Bell et al. showed that TiO_2_/rGO nanocomposite has a higher efficiency than pristine TiO_2_. This is because RGO plays an important role as a highly conductive intraparticle charge transport network within the film, leading to a fourfold increase in the electron lifetime [54]. Another report showed that the incorporation of RGO with TiO_2_ as a photoanode in a dye-sensitized solar cell (DSSC) improved the electron transport and reduced the charge recombination, yielding better performance compared with that of a device with a pure TiO_2_ photoanode [55]. Sookhakian et al. reported that ZnS–RGO composites showed an improvement in photocurrent generation with reduction of charge transfer resistance and charge recombination compared to pure ZnS NPs [56]. Moreover, Lei et al. found that the electron capture and transfer ability of graphene could enhance the photoelectric characteristics of the nanocomposites via a comparison between Gr–CdS nanocomposites and pure CdS NPs [57]. Similarly, the performance of the CIGS device could be further enhanced using the Gr–CdS nanocomposite. Enhanced photocurrent density for the Gr–CdS composite compared with the pure CdS was also confirmed by the water splitting study reported in Section 3.2.2.

#### 3.2.2. Water Splitting 

**Figure 9:**
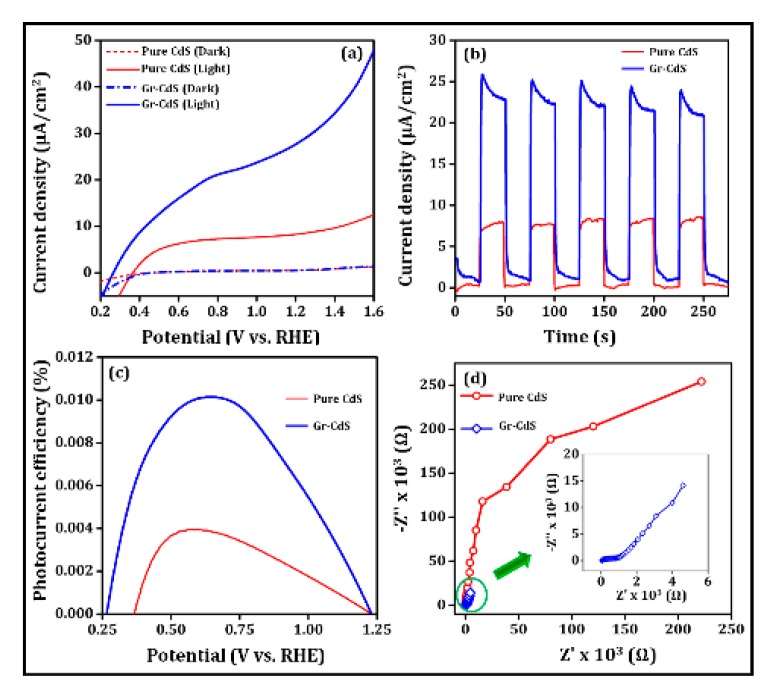
(**a**) Photocurrent density as a function of applied potential (V vs. RHE) under illumination and in the dark, (**b**) transient photocurrent response of Gr–CdS and pure CdS during periodic on–off light cycles, (**c**) photoconversion efficiency against an applied potential (V vs. RHE), and (**d**) EIS spectra of Gr–CdS and pure CdS.

To evaluate the PEC water splitting efficiency of the CdS NPs and Gr–CdS nanocomposite, the photocurrent density was recorded under an applied potential in 0.5 M Na_2_SO_4_ electrolyte, as shown in Figure 9a. The polarization curve of the photoanodes showed that the photocurrent density of the Gr–CdS sample was threefold higher than the pure CdS NPs, which ultimately resulted in more efficient water splitting. These results also agree well with the enhanced J_SC_ of the CIGS solar cell with the Gr–CdS composite compared to the cell with pure CdS NPs. The improved PEC activity of the Gr–CdS nanocomposite is due to the interfacial charge transfer process, in which graphene plays the important role of accepting the produced electrons from the conduction band of CdS, ultimately reducing the recombination rate between holes and electrons [58]. The PEC performance of the Gr–CdS nanocomposite and pure CdS NPs photoanodes was then monitored by conducting time-dependent chronoamperometry measurements, in which multiple visible light on–off cycles were applied to the photoanodes. The photocurrent densities of the pure CdS NPs and Gr–CdS nanocomposite photoanodes at a bias potential (1 V vs. RHE) during on–off illumination cycles with an interval time of 20 s are shown in Figure 9b. The background currents of the photoanodes are almost zero under the dark condition. When they are illuminated, a photocurrent is instantly generated; the photocurrent exhibits a spike resulting from the rapid photoresponse upon light excitation and then enters a steady state. The photocurrent decays to zero once the light is switched off [59]. The current density of the device with the Gr–CdS nanocomposite anode was three times higher than that of the device with the pure CdS NPs film. In Table 3, we compare our photocurrent density results with recent results in the literature.

The solar-to-hydrogen (STH) conversion efficiency (η) was calculated using the following equation:

(2) $$\eta =J_{P}\;\left(\;\frac{1.23-\;V_{app}}{I_{0}}\;\right)\;\times \;100\%$$

where *J_p_* is the photocurrent density (mA/cm^2^) at the applied bias and *I_0_* is the incident light intensity. The *V_app_* is defined as *V_mea_* − *V_aoc_* in which *V_aoc_* is the electrode potential (vs. SHE) of the working electrode under an open bias condition, and V*_mea_* is the potential (vs. RHE) of the same working electrode [60]. Figure 9c shows the plot of the photoconversion efficiency vs. the applied potential (V vs. RHE). The STH efficiency of the Gr–CdS composite reached a maximum of 0.01% at an applied potential of 0.625 V (vs. RHE), which is remarkably higher than the STH efficiency of the pure CdS NPs under this low-bias condition. The improved charge transport in the Gr–CdS nanocomposite photoanodes is further demonstrated by the EIS results. Nyquist plots of the prepared Gr-CdS composite and pure CdS NPs photoanodes are presented in Figure 9d, where the impedance spectrum consists of a semicircular arc. A semicircle with a smaller diameter indicates better charge transport behavior [61]. The impedance spectrum of the Gr–CdS nanocomposite photoanodes showed a typical semicircle with a smaller radius compared with that of the CdS electrode, indicating enhanced separation and transport of the generated carriers, and resulting in a higher photocurrent response. As listed in Table 3, the prepared material exhibited a relatively high photocurrent response compared to similar photoanodes in a neutral electrolyte under low bias conditions.

## 4. Conclusions

A Gr–CdS nanocomposite was successfully prepared using a simple chemical solution method, and its formation and primary material properties were precisely characterized by several analysis techniques including EDS, XPS, XRD, Raman spectroscopy, and TEM. It was also demonstrated that a CIGS solar cell using the Gr–CdS nanocomposite as a buffer layer showed higher J_SC_ values, V_OC_ values, and efficiency than a cell using pure CdS NPs. Furthermore, the Gr–CdS composite exhibited excellent performance in the production of hydrogen as a clean and storable source of energy via photoelectrochemical water splitting.

## Figures and Tables

**Table 1:** Applications of graphene or its derivatives in solar cells and reported device performance.

Solar Cell Type	Graphene Type	Graphene Deposition Method	Role of Graphene	Photovoltaic Parameters				Ref.
				J_sc_(mA/cm^2^)	V_oc_(V)	FF(%)	Efficiency(%)	
CdTe	Gr	CVD	Front electrode	22.9	0.430	42.0	4.17	[19]
	B:Gr	Solution method	Back electrode	22.0	0.685	52.2	7.86	[20]
	Bi:RGO	Hummers method		26.2	0.787	63.7	13.2	[21]
CIGS	Au:Gr	CVD	Front electrode	32.4	0.601	69.1	13.5	[22]
	Gr		Back electrode	28.8	0.531	64.7	9.91	[23]
			Mo/Gr back electrode	-	-	-	-	[24]
PSC	GO	Hummers method	Buffer layer	20.9	1.04	66.0	14.4	[25]
	Gr	CVD	Front electrode	18.6	1.04	59.4	11.5	[26]
	Gr		Blocking layer	21.1	1.09	68.2	15.7	[27]
n-Si	N-doped Gr		Front electrode	30.9	0.490	41.2	6.24	[28]
	GO	Hummers method	Antireflection	38.4	0.512	53.0	10.6	[29]
DSSC	Gr	CVD	Front electrode	7.80	0.630	40.0	2.0	[30]
	SnS@RGO	Solution method	Counter electrode	18.9	0.705	57.9	8.21	[31]
OSC	Gr doped with GQDs and Ag NWs	CVD	Front electrode	10.4	0.592	59.3	3.66	[32]
	Gr			4.73	0.480	52.0	1.18	[33]
GaAs	Gr	Simulation	Formation of Schottky junction	2.14	0.350	69.7	5.3	[34]

(*) Note: CVD (chemical vapor deposition), RGO (reduced graphene oxide), CIGS (Cu(In, Ga)Se_2_), GO (graphene oxide), PSC (perovskite solar cell), DSSC (dye-sensitized solar cell), and OSC (organic solar cell).

**Table 2:** Elemental composition of pure CdS and Gr–CdS nanocomposite powders measured by EDS.

Material	Cd (at%)	S (at%)	C (at%)	Cd/S (-)
Pure CdS	47.6	52.4	0.0	0.91
Gr-CdS	10.7	11.5	77.8	0.93

**Table 3:** Comparison of our photocurrent density results with recently reported results.

Material Structure	Preparation Method	Experiment Condition	Electrolyte	Current Density (μA/cm^2^)	Ref.
Gr–CdS	Hydrothermal	180 °C, 40 h	0.5 M Na_2_SO_4_	55	[62]
	Hydrothermal	140 °C, 24 h	0.2 M Na_2_SO_4_	12	[35]
	Chemical precipitation	25 °C, 48 h	0.25 M Na_2_S and 0.35 M Na_2_SO_3_	120	[63]
	Hydrothermal	200 °C, 6 h	0.24 M Na_2_S and 0.35 M Na_2_SO_3_	45	[64]
	Hydrothermal	120 °C, 12 h	0.1 M H_2_SO_4_	15	[57]
	Hydrothermal	180 °C, 12 h	0.1 M Na_2_SO_4_	0.01	[65]
N-graphene/CdS	Hydrothermal	25 °C, 48 h	0.5 M Na_2_SO_4_	40	[66]
RGO–CdS	Hydrothermal	120 °C, 48 h	--	9	[67]
ITO/Gr–CdS:Mn	Hydrothermal	200 °C, 12 h	0.1 M Na_2_SO_4_	15	[68]
Gr–CdS–PANI-6	Hydrothermal	70 °C, 6 h	0.1 M KCl	0.4	[69]
Gr-–CdS	Chemical precipitation	25 °C, 2 h	0.5 M Na_2_SO_4_	40	This work
